# A Compact Planar Monopole UWB MIMO Antenna for Short-Range Indoor Applications

**DOI:** 10.3390/s23094225

**Published:** 2023-04-23

**Authors:** Shanmugam Kolangiammal, Loganathan Balaji, Miroslav Mahdal

**Affiliations:** 1 Department of Electronics and Communication, Vel Tech Rangarajan Dr. Sagunthala R&D Institute of Science and Technology, Chennai 600062, India; 2 Department of Electronics and Communication, Faculty of Engineering and Technology, SRM Institute of Science and Technology, Kattankulathur 603203, India; 3 Department of Control Systems and Instrumentation, Faculty of Mechanical Engineering, VSB-Technical University of Ostrava, 17. Listopadu 2172/15, 70800 Ostrava, Czech Republic

**Keywords:** directive gain, envelope correlation coefficient (ECC), monopole, multiple input multiple output (MIMO), ultra-wideband antenna (UWB)

## Abstract

A compact, four-element planar MIMO (Multiple Input, Multiple Output) antenna that operates in an ultra-wideband is presented for diversity application. The orthogonal position of the unit cells replicates the single antenna thrice, thereby decreasing mutual coupling. A UWB MIMO antenna measuring 35 × 35 × 1.6 mm^3^ is built using a microstrip line (50 Ω impedance) on an FR4 substrate having a thickness of 1.6 mm. The ground plane and radiator of this antenna are adjusted in several ways to bring it within its operating constraints between the frequencies of 3.1 GHz and 10.6 GHz. This technique makes the antenna small and covers the entire ultra-wideband (UWB) frequency range. The NI USRP was used to test the proposed MIMO antenna to determine its real-time performance. Based on the computed results, we conclude that this proposed antenna has outstanding characteristics in terms of performance and is suitable for wireless ultra-wideband indoor communication and diversity utilization with a small size.

## 1. Introduction

UWB refers to an ultra-wideband with a frequency ranging from 3.1 to 10.6 GHz. The bandwidth of 7.5 GHz for UWB communications was authorized by the Federal Communications Commission (FCC) in 2002 for use in a variety of commercial applications [1]. MIMO technology is crucial to current 5G communications due to increased bandwidth demands, faster data rates, and applications for smart homes and RFID. UWB systems are created to improve short-range high-data-rate applications, radar imaging, automotive transmission communications, and system channel capacity [2]. To achieve great isolation, the two radiators’ radiation patterns are orthogonally polarized with respect to one another, and the feeds of the two antennas are positioned similarly [3]. Two long ground stubs that operate as parasitic monopoles and a short ground strip are utilized to increase isolation and bandwidth [4]. The antenna comprises a radiation patch connected by a strip that is positioned below the patch. The strip serves as an impedance transformer in addition to offering a second communication path, resulting in effective isolation [5]. A brand-new fence-type decoupling structure that offers excellent isolation in the UWB region has been proposed [6]. The antenna’s impedance bandwidth at higher UWB bands is extended by the parasitic resonance of a ground stub [7]. Mutual coupling between the radiating patches is reduced using a neutralization line [8]. To keep the antenna’s size to a minimum, side-by-side complementary split-ring elements are employed as the primary radiator [9]. Increased isolation is achieved by the presence of a T-shaped stub of the UWB MIMO antenna between the element of the antenna and the surface [10]. The use of a symmetrical curved-I-shaped DGS has been shown to increase the bandwidth and decrease mutual coupling [11]. To obtain higher isolation, protruded ground and parasitic elements are added [12]. The single antenna is a patch with a bow-tie slot in the center and slits on the sides, whereas each MIMO antenna is a two-element array supplied with a corporate feeding network. To enhance the performance of the antenna, the vertical and horizontal slots are integrated as a defected ground structure (DGS). To further improve the isolation, a slotted zig-zag decoupling structure is etched from edge to edge on the top surface [13]. To attain high gain and efficiency, a single-element antenna based on the Murkowski fractal shape is used as a defected ground structure (DGS) [14]. The use of the floating parasitic element enhances isolation at higher frequencies [15]. The use of two sequential iterations of defected ground structure (DGS) reduces the mutual coupling between the closely spaced antenna components [16]. The orthogonal polarization technique is used to streamline the design process and minimize mutual couplings [17]. A self-feeding Janus metasurface SF-MS is used to manipulate the incident EM waves and emit radiated waves [18].

The main and primary contribution of this paper is the thorough magnified presentation of four compact ultra-wide-bandwidth MIMO antennas with isolation between ports having more than 35 dB that satisfy the criteria and operates well between 3.1 and 10.6 GHZ. The rest of this work is structured as follows. The design process for a single antenna and four-element MIMO antennas are thoroughly described in Section 2 of this work. The findings regarding the antenna’s measurements are presented and discussed in Section 3. Section 4 describes an antenna test using USRP in an indoor environment for MIMO applications. In Section 5, conclusions are provided.

## 2. Proposed Antenna Design

### 2.1. Single-Cell Antenna Design

**Figure 1:**
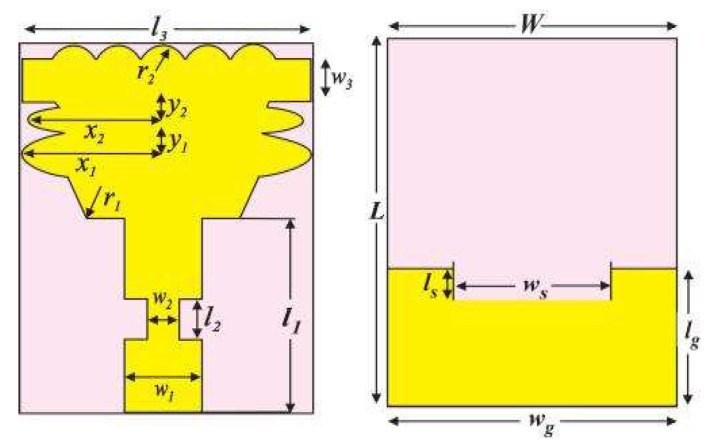
Unit-Cell Antenna, the Front and Rear View.

The proposed unit-cell antenna, based on an FR4 substrate, has a microstrip feed. The FR4 substrate has a 4.3 relative permittivity ($\varepsilon_{r}$) and a 0.025 loss tangent. The thickness and the permittivity are the criteria that should be selected for the substrate; thus, these characteristics clearly make an impact on bandwidth. FR4 has advantages over having electrical features, with prices being affordable and reasonable, provided there is availability. 18 mm × 16 mm × 1.6 mm was the volume that the planned antenna occupied. Table 1 shows the results of the measurements for various parameters. Figure 1 displays the back and front views of the final proposed antenna.

The antenna is made up of a radiator at the front that combines two ellipses, two hexagons, two rectangles, five circles, and a tapering microstrip feed, along with a defective rectangular ground object at the back. The novelty radiator’s geometric specs are as follows: Atop an ellipse with a major axis $x_{1}=7\;mm$ and minor axis $y_{1}=1\;mm$, which furthermore is mounted on a different ellipse with major axis $x_{2}$ = 7.5 mm and minor axis $y_{2}=1.4\;mm$, are five neighboring tangential circles, each measuring 1.5 mm radius. Furthermore, 2 hexagons with a 2 mm radius, positioned alongside the edges of a 3 × 6 ${mm}^{2}$ rectangle, are the setup of the entire arrangement. Moreover, to minimize mutual coupling as well as improve impedance-matching potential, a 15 × 2 ${mm}^{2}$ thin rectangle is employed while passing through the arrangement of 5 circles positioned on top.

**Figure 2:**
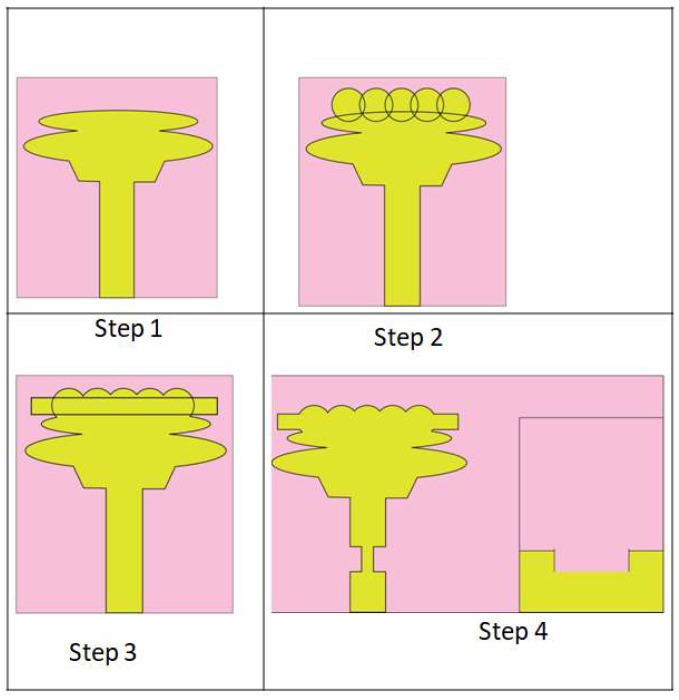
Evolution of monopole UWB Unit-cell Antenna.

**Figure 3:**
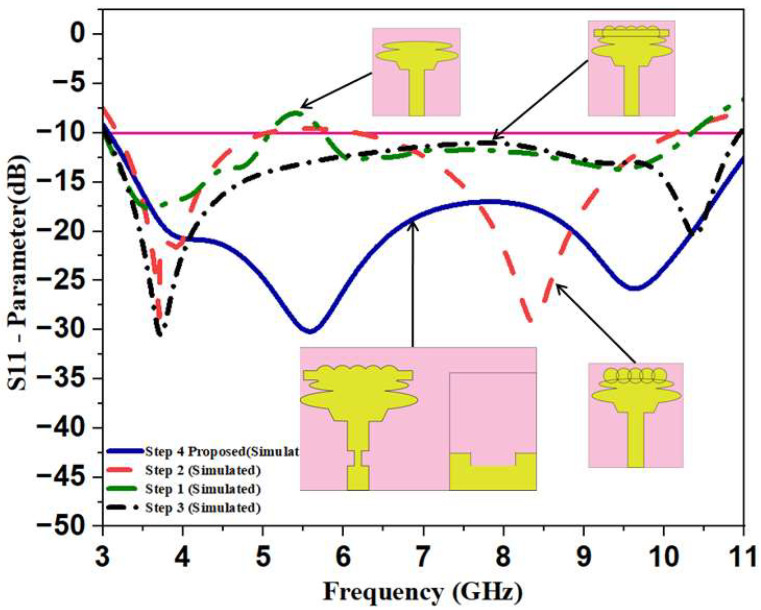
Simulated S-Parameter S11 during Evolution Stages.

Various stages of evolution or processing are carried out in order to arrive at the final stage of the product or design of the single-unit-cell antenna. At every stage, the S-parameter of the simulated antenna cell is evaluated, and suitable modifications are incorporated into the unit-cell design to improve the S-parameter. The final design of the unit-cell antenna is achieved over four stages of evolution, as shown in Figure 2. The simulated S parameter during evolution stages is displayed in Figure 3. The antenna designed in Step 1 consists of a low-profile, compact-sized microstrip-line-fed ellipse-shaped patch (refer to Step 1 in Figure 2); however, it has a poor impedance, which is the green curve shown on the graph in Figure 3. It only works when the conditions are satisfied and the ultra-wideband is at a frequency ranging from 3.1 to 10.6 GHz. In evolution Step 2, five circle-shaped radiators were integrated into a microstrip-fed elliptical-shaped radiator to increase impedance bandwidth, as shown in Step 2 in Figure 2. In this modification, lower UWB frequencies are well covered, but upper UWB frequencies are not covered well. In Step 3, we add a rectangular radiator to increase impedance bandwidth. The resulting impedance-matching curve is shown in red color. It can be seen from the curve that the impedance matching varies widely across the frequency range. The final evolution is carried out to confine the variations in the impedance matching by using a tapered feed and defected ground structure technique, as shown in Step 4 of Figure 2. The corresponding curve is shown in blue color in Figure 3. The following equation shows how the Q factor rises, and a high-level inductive reactance results from the intersection of ellipses, hexagons, rectangles, and circles.

(1) $$Q=\frac{\omega L}{R}=\frac{X_{L}}{R}$$

**Figure 4:**
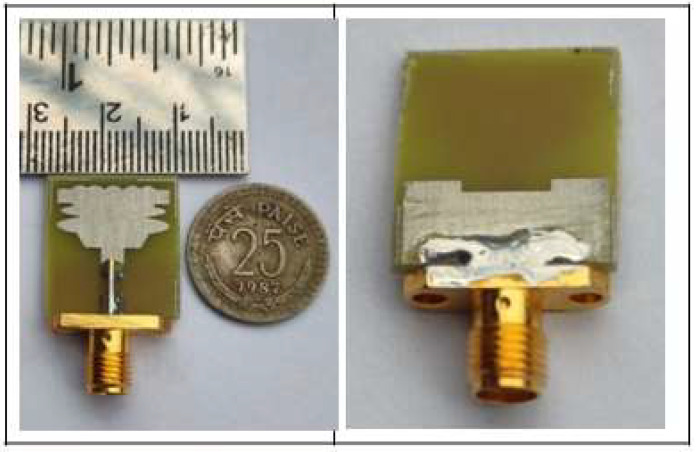
Fabricated Single UWB Antenna.

The ground (surface) plane is constructed with a rectangle-shaped slot that is 7 × 16 ${mm}^{2}$ in size and is utilized to increase bandwidth and impedance matching. The slot’s inclusion led to a significant boost in bandwidth by reducing the capacitance influence between the surface plane and the radiator. To increase the current flooding and bring it close to the radiator to permit the necessary impedance bandwidth, the feed line, which has dimensions of 9 × 3 ${mm}^{2}$, is tapered. Both the inductive reactance and the capacitance reactance were canceled out by the change in the other. The antenna then began to operate like a totally resistive load. A revolutionary elliptical radiator is a clever way to create a small-scale UWB MIMO antenna. Figure 4 shows a fabricated single UWB antenna. 

**Figure 5:**
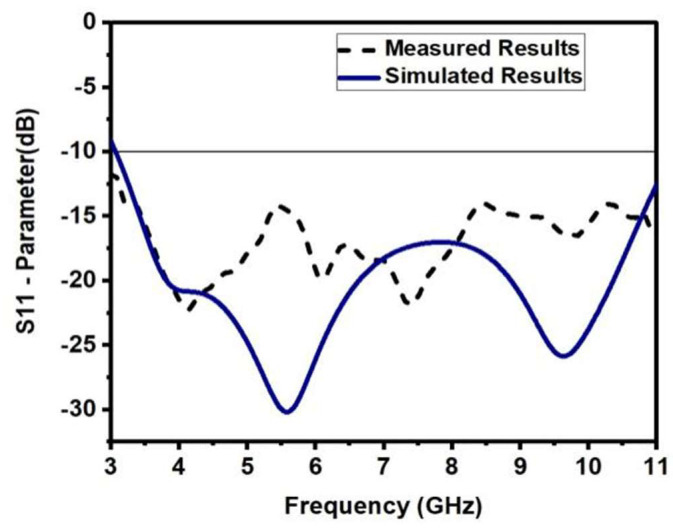
Simulated and Measured S11 Parameter of the Monopole UWB antenna.

The scattering characteristics (S11) of the antenna are shown in Figure 5 to be below −10 dB for both simulated and measured results across the specified bandwidth. The measured S-parameters differ from those simulated due to the fabrication tolerance and other aspects of the SMA connector. 

### 2.2. MIMO Antenna Design

**Figure 6:**
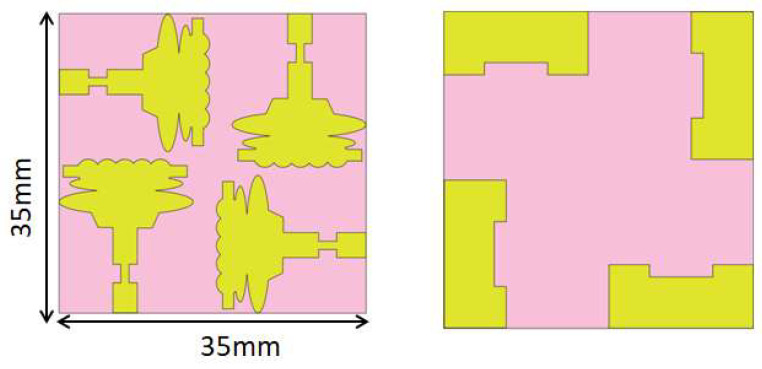
Sketch of UWB MIMO Antenna, the Front and Rear View.

**Figure 7:**
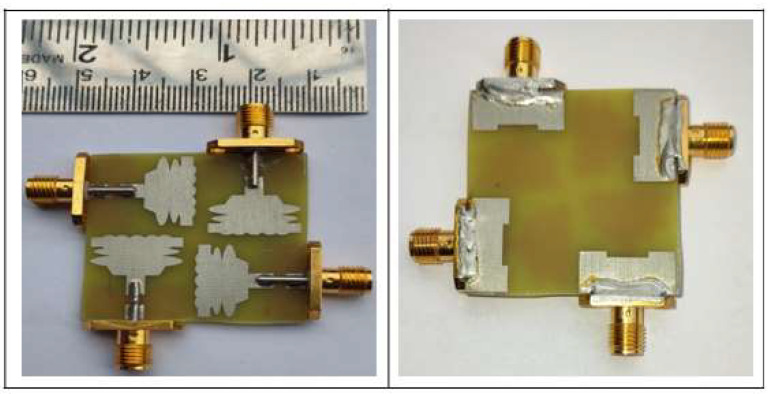
Fabricated UWB MIMO Antenna, the Front and Rear View.

**Figure 8:**
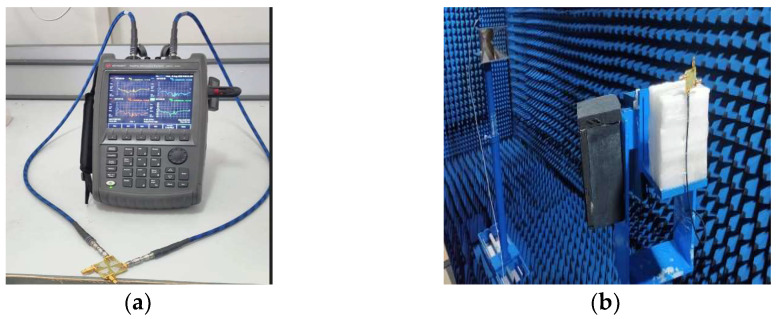
Measuring Proposed Ultra-Wideband MIMO Antenna Parameter in (**a**) Vector Network Analyzer, (**b**) Anechoic Chamber.

To create a multi-port antenna with good isolation, the single-cell antenna is to be duplicated and arranged such that it forms an orthogonal pattern. On a single plane, all radiating components are organized. The four-port antenna’s overall dimensions are 35 × 35 × 1.6 ${mm}^{3}$, where $h_{s}$ stands for the substrate’s height. The simulated and fabricated UWB MIMO antenna is shown in Figure 6 and Figure 7, respectively. Figure 8a,b show the measurement setup for the constructed antenna in the vector network analyzer and anechoic chamber.

## 3. Results and Discussion

By performing parametric analysis and optimization, the proposed antenna is structured and developed using CST Microwave Studio Software. The proposed MIMO/Diversity antenna is measured using an Anritsu MS2703 Vector Network Analyzer.

### 3.1. Mutual Coupling

Four antennas that appear to be quite close to one another are included in the monopole ultra-wideband Multiple-In Multiple-Out antenna. As a result, interference from one antenna’s radiation with the others would occur.

**Figure 9:**
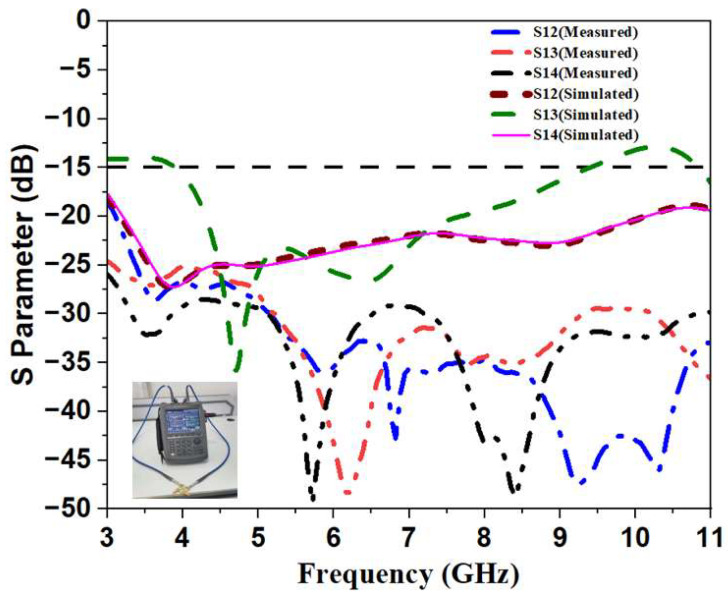
Simulated and Measured S-Parameter Isolation of Monopole Ultra-Wideband MIMO Antenna.

By building the redesigned ground structures, the mutual coupling is decreased, and identical antennas are positioned orthogonally. For the full bandwidth range, this antenna is designed such that it decreases mutual coupling to lower than −35 dB. The results of the S-Parameter simulation and measurement for the monopole ultra-wideband MIMO antenna are shown in Figure 9. It is clear that the etched surface plane with a slot provides good isolation and impedance matching. As a result, the desired outcome was attained.

### 3.2. Envelope Correlation Coefficient (ECC)

**Figure 10:**
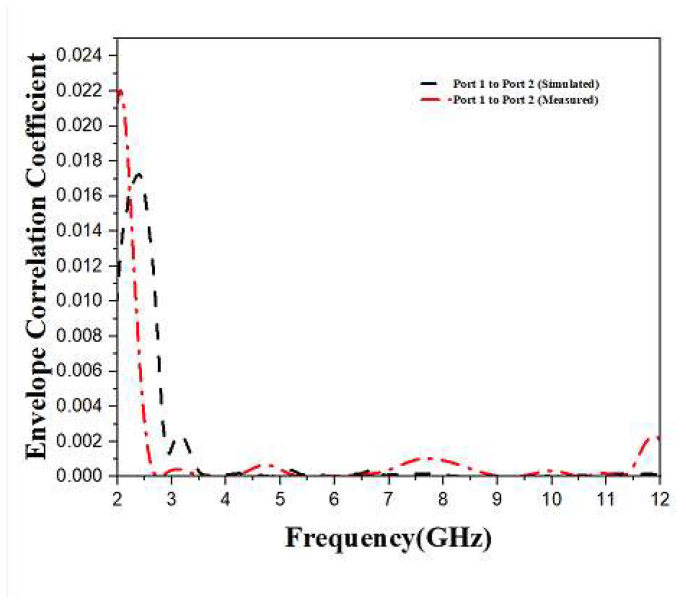
Simulated and Measured ECC of Monopole Ultra-Wideband MIMO Antenna.

Four antennas that appear to be quite close to one another are included in the correlation that exists between the radiating objects. This correlation is the most significant diversity characteristic of the Multiple-In Multiple-Out antenna. The degree of independence relating to the radiation patterns of two antennas is described with the help of ECC. Usually, the radiation patterns of the two antennas are completely not dependent on each other. The value of ECC must be 0; however, its real value must not be more than 0.5. The calculation of the envelope correlation coefficient is carried out by substituting the S-Parameter in Equation (2) [19,20]. Through Figure 10, it is evident that the value of ECC is below 0.001. Furthermore, this value can be considered a good envelope correlation coefficient.

(2) $$ECC=\frac{{\left|S_{11}^{*}S_{12}+S_{21}^{*}S_{22}\right|}^{2}}{\left(1-{\left|S_{11}\right|}^{2}-{\left|S_{21}\right|}^{2}\right)\left(1-{\left|S_{22}\right|}^{2}-{\left|S_{12}\right|}^{2}\right)}$$

### 3.3. Diversity Gain (DG)

**Figure 11:**
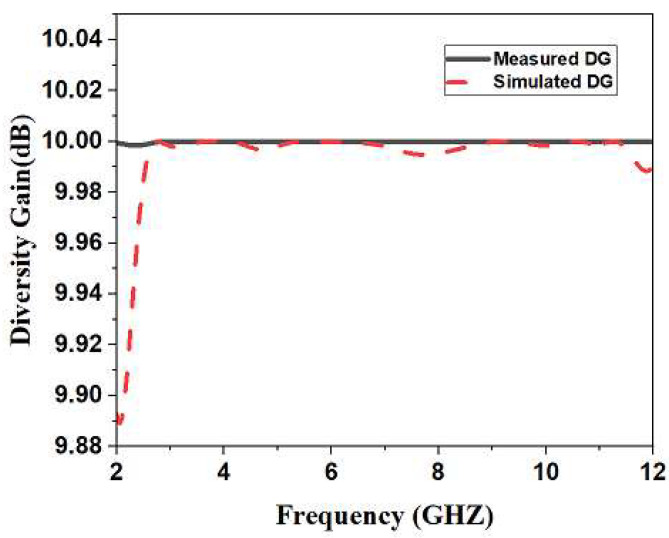
Simulated and Measured Diversity Gain of Monopole Ultra-Wideband MIMO Antenna.

In order to retain the dependency of the wireless communication system along with high-quality output, it is necessary to maintain the diversity gain of the monopole ultra-wideband Multiple-In Multiple-Out antenna at its maximum. Ideally, 10 dB is the best value. The diversity gain value for the suggested antenna can be derived using the formula in Equation (3) [21]. We can see from Figure 11 that the greatest diversity gain increase is 9.999 dB.

(3) $$DG=10\sqrt{1-{ECC}^{2}}$$

### 3.4. TARC

TARC represents the ratio of the incident power to the squared value of the reflected power. Equation (4) represents the TRAC ratio of the N port antenna.

(4) $$\Gamma_{a}^{t}=\frac{\sqrt{\sum_{i=1}^{N}{\left|y_{i}\right|}^{2}}}{\sqrt{\sum_{i=1}^{N}{\left|x_{i}\right|}^{2}}}.$$

where *x_i_* denotes incident signals; *y_i_* denotes reflected signals. 

The scattering matrix pattern of a 2 *×* 2 arrangement of the antenna is shown in Equation (5).

(5) $$\left[\begin{array}{c} y_{1}\\ y_{2} \end{array}\right]=\left[\begin{array}{cc} s_{11} & s_{12}\\ s_{21} & s_{22} \end{array}\right]\left[\begin{array}{c} x_{1}\\ x_{2} \end{array}\right].$$

Every excitation signal’s phase in the Multiple-In Multiple-Out antenna is arbitrary. Before a signal reaches a receiver, the propagation environment helps to randomize the signal phases further. As a result, the MIMO channel signal is regarded as having an independent, identical distribution with a random phase. Equations (6) and (7) can be used to express reflected signals. The sum or difference of the Gaussian random variables produces the Gaussian values [21].

(6) $$y_{1}=S_{11}x_{1}+S_{12}x_{2}=S_{11}x_{0}e^{j\theta 1}+S_{12}x_{0}e^{j\theta 2}=x_{1}\left({S_{11}+S}_{12}x_{0}e^{j\theta }\right).$$

(7) $$y_{2}=S_{21}x_{1}+S_{22}x_{2}=S_{21}x_{0}e^{j\theta 1}+S_{22}x_{0}e^{j\theta 2}=x_{1}\left({S_{21}+S}_{22}x_{0}e^{j\theta }\right)$$

Thus, TARC is explained in the following Equation (9).

(8) $$\Gamma_{a}^{t}=\frac{\sqrt{{{(\left|x_{1}(S_{11}+{S_{12}e}^{j\theta })\right|}^{2}+\left|x_{1}({S_{21}+S_{22}e}^{j\theta })\right|}^{2}}}{\sqrt{{2\left|x_{1}\right|}^{2}}},$$

(9) $$\Gamma_{a}^{t}=\frac{\sqrt{{{(\left|\left(S_{11}+{S_{12}e}^{j\theta }\right)\right|}^{2}+\left|\left({S_{21}+S_{22}e}^{j\theta }\right)\right|}^{2}}}{\sqrt{2}}.$$

**Figure 12:**
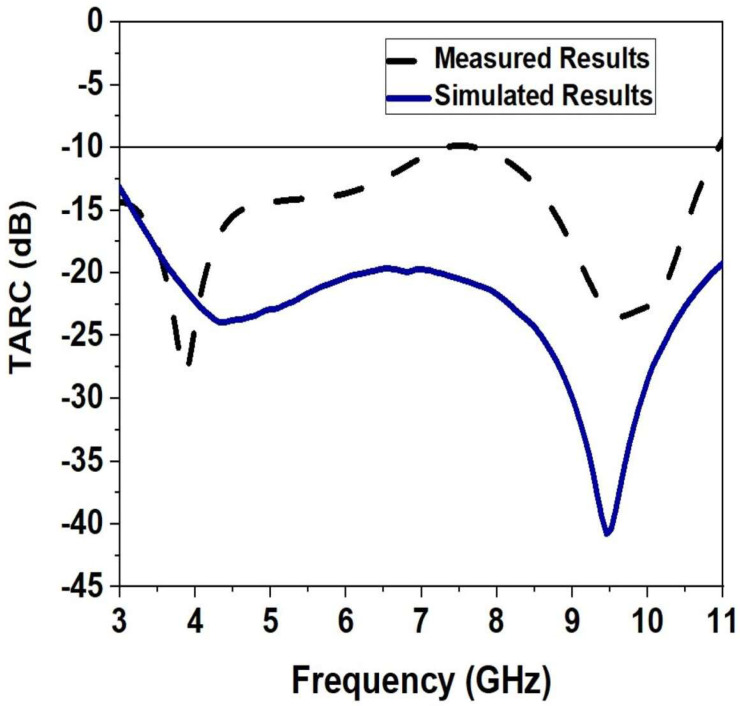
Simulated and Measured TARC of the Monopole Ultra-Wideband MIMO Antenna.

The return loss of the entire proposed MIMO antenna is known as TARC. TARC was first proposed to assess a multiport radiator’s performance. Because it takes into account mutual coupling, port matching, and the impact of the random phases of incoming signals into each antenna element, TARC has recently been found to be useful as a MIMO metric for antenna systems. This is because it describes the performance in a more realistic situation of a communications channel. TARC is the sole MIMO parameter that takes into account the unpredictable phases of incoming signals, which can have a significant impact on MIMO array behavior in certain circumstances. Over the whole working frequency band, TARC is less than −10 dB. The obtained TARC is shown in Figure 12. 

### 3.5. MEG

The definition of MEG is the segregation of power received by an isotropic antenna with power obtained from a diversity antenna [22,23]. The MEG value for the Multiple-In Multiple-Out antenna system is obtained through the relationships given below. Equation (10) can be used to calculate *MEG*1 and *MEG*2.

(10) $${MEG}_{i}=0.5\left(1-\sum_{j=1}^{N}{S_{ij}}^{2}\right)i=1,2$$

**Figure 13:**
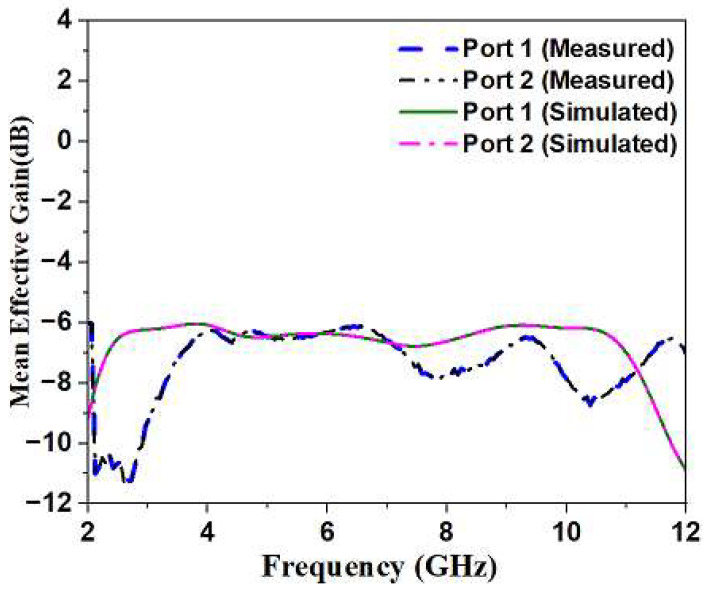
Simulated and Measured MEG of Monopole Ultra-Wideband MIMO Antenna.

Figure 13 closely reveals that *MEG*-1 and *MEG*-2 are identical due to the enhanced total performing capacity of the proposed radiator.

### 3.6. CCL

The highest message rate at which a message may be sent across a communication channel without experiencing any losses is referred to as channel capacity loss. Equation (11) [24] can be used to express it, and the following components can be used to estimate it.

(11) $$C_{Loss}=-\log_{2}det\left(\Psi^{R}\right),$$

(12) $$\psi^{R}=\left[\begin{array}{cc} \psi_{11} & \psi_{12}\\ \psi_{21} & \psi_{22} \end{array}\right],$$

where

$$\Psi_{11}=1-\left({\left|S_{11}\right|}^{2}+{\left|S_{12}\right|}^{2}\right),$$

$$\Psi_{21}=1-\left({\left|S_{22}\right|}^{2}+{\left|S_{21}\right|}^{2}\right),$$

$$\Psi_{12}=-\left({S^{*}}_{11}S_{12}+{S^{*}}_{21}S_{22}\right),$$

$$\Psi_{21}=-\left({S^{*}}_{22}S_{21}+{S^{*}}_{12}S_{11}\right).$$

**Figure 14:**
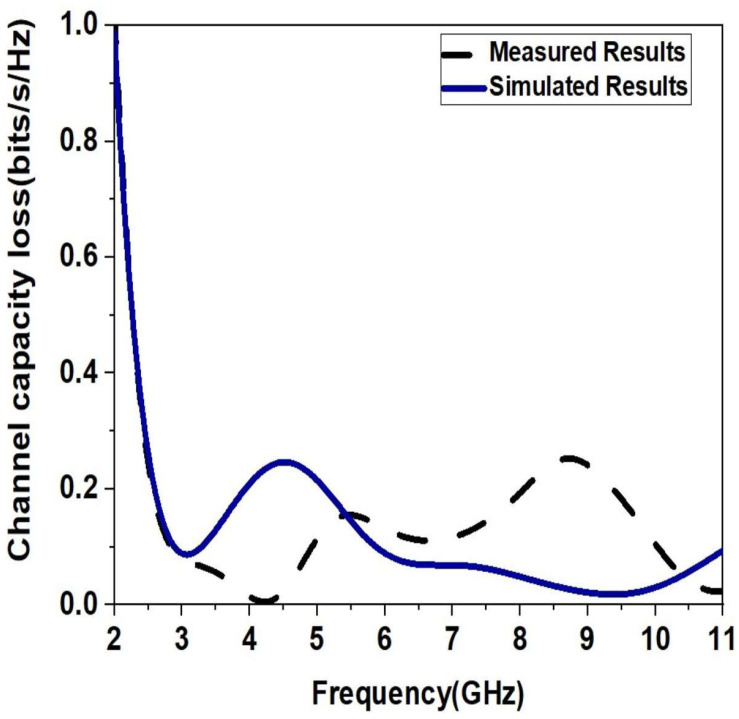
Simulated and Measured CCL of the Monopole Ultra-Wideband MIMO Antenna.

The value of CCL value must be less than 0.4 bits/S/Hz in general. Figure 14 clearly explains the CCL value of the designed MIMO antenna in relation to the operating frequency limit until acceptable CCL values are reached.

### 3.7. Radiation Patterns

**Figure 15:**
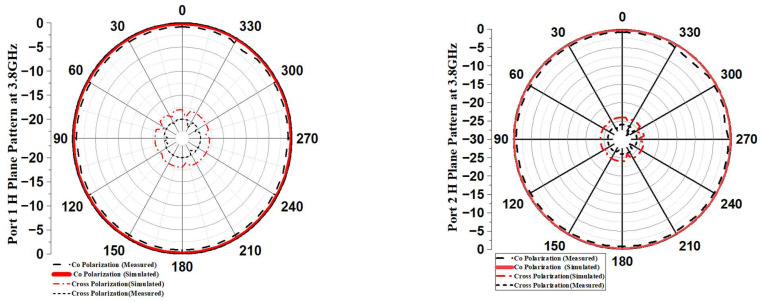

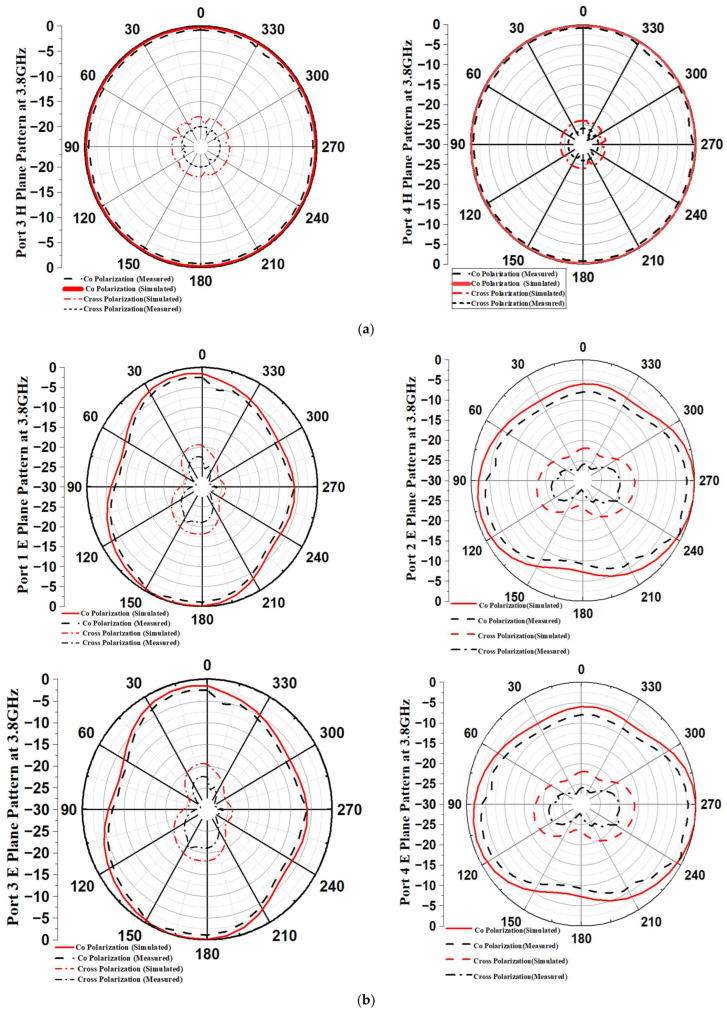
(**a**) H-Plane Pattern of Port 1, Port 2, Port 3, and Port 4 of designed Ultra-Wide band MIMO Antenna at 3.8 GHz. (**b**) E-Plane Pattern of Port 1, Port 2, Port 3 and Port 4 of designed UWB MIMO at 3.8 GHz.

The proposed antenna’s simulated and measured E-plane and H-plane radiation patterns at 3.8 GHz frequency are illustrated in Figure 15a,b. 

With the help of this pattern, it is possible to demonstrate how the designed MIMO antenna exemplifies pattern variety in the 3.8 GHz frequency range. The Port 1 and Port 3 patterns are moved 180 degrees in relation to one another, and Ports 2 and 4 are turned 180 degrees in relation to one another. The proposed antenna’s E-plane radiation pattern is shaped like a dumbbell, while the H-plane radiation pattern is omnidirectional [25,26]. A stable radiation performance is indicated by low cross-polarizations in both planes and a difference between co-polar and cross-polar radiation patterns higher than 15 dB.

Table 2 presents the quantitative comparison of the proposed antenna design with the antenna designs reported in the literature. The proposed antenna is believed to have acquired significant results with respect to CCL below 0.4 bits/s/Hz, 0.001 ECC, TARC below −10 dB, 9.999 dB DG, and MEG less than 3 dB.

## 4. Antenna Test Using USRP at an Indoor Environment for MIMO Applications

**Figure 16:**
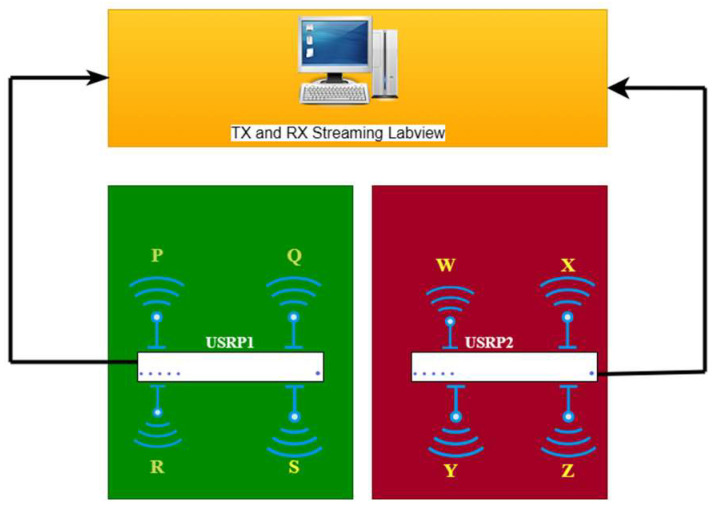
Block Diagram of Indoor Antenna Test with the Help of USRP.

**Figure 17:**
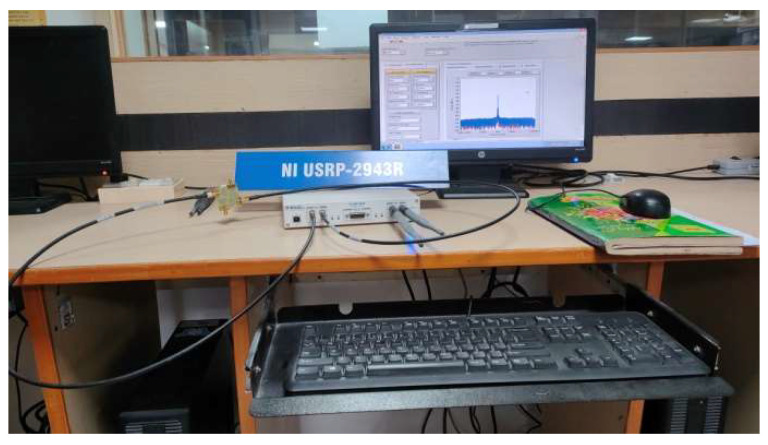
Image of Real-time Indoor Base Station Setting with the Proposed MIMO Antenna.

The investigation of the designed ultra-wideband MIMO compact antenna utilized the NI 2943R (USRP) and NI 802.11 framework in an indoor setting to confirm the proposed MIMO antenna’s real-time transmitting and receiving capabilities. The National Instruments (NI) Universal Software Radio Peripheral (USRP) is a technology-driven RF transceiver operated for software-defined radio R&D (SDR). For communications purposes, USRP NI transceivers are capable of transmitting and receiving radio frequency signals in a number of bands. The indoor setting is composed of an antenna feed system, two NI USRPs, and a received power monitor. For verification of real-time measurements, consideration should be given to signal reception power (RSRP) parameters [31,32]. The average received power of a single RS resource element is called the RSRP. The proposed MIMO antenna consists of two compact four-port UWB MIMO antennas that are coupled to USRP and broadcast signals in a variety of states. A block presentation of the proposed MIMO antenna using the internal transmission and receipt of NI USRP is shown in Figure 16. The Indoor Base station setting with the proposed MIMO antenna is shown in Figure 17.

To test the MIMO antenna’s real-time performance, three alternative states are used.

State 1: Radiators W, X, Y, and Z are considered to be the receivers, whereas radiators P, Q, R, and S are considered to be the transmitters. 

State 2: Antenna radiators P, Q, W, and X are considered transmitters, whereas radiators R, S, Y, and Z are considered receivers.

State 3: The antenna radiators W, X, Y, and Z are considered to be transmitters, whereas P, Q, R, and S are considered to be receivers. 

**Figure 18:**
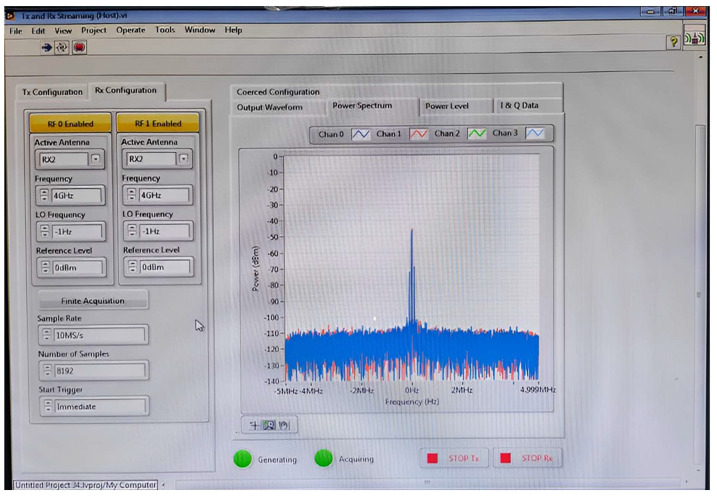
RSRP Measurement against RX and TX Streaming Lab View.

**Figure 19:**
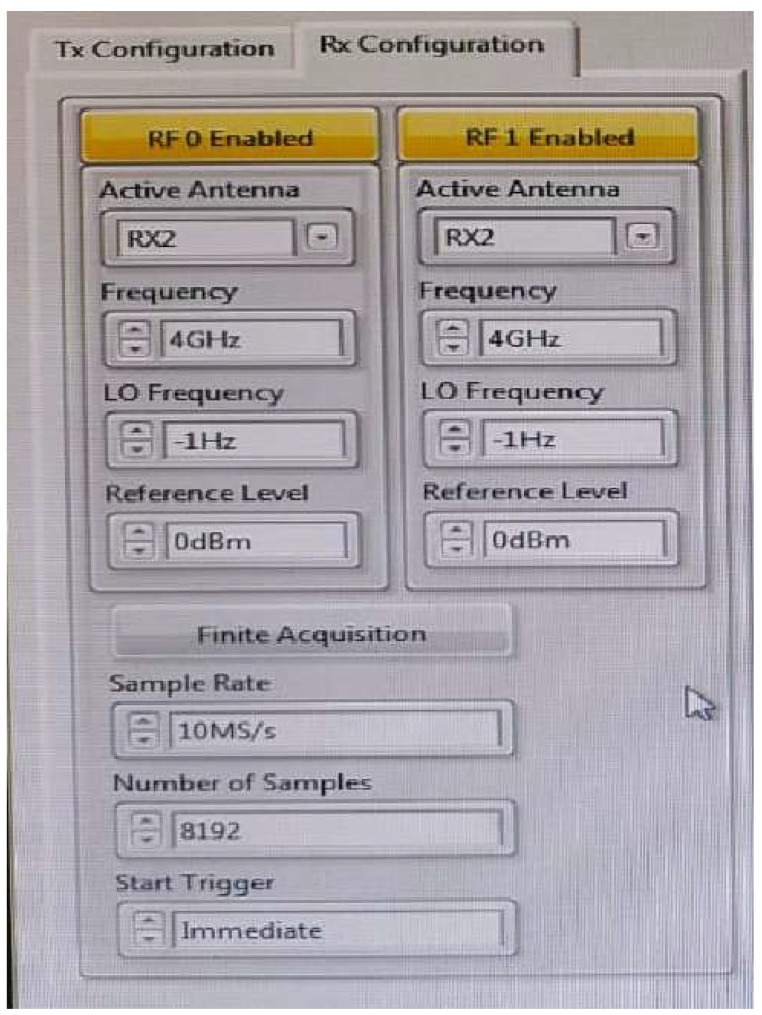
RSRP Measurement against RX and TX Streaming Lab View (zoomed view).

Using the USRP 2943R, Figure 18 and Figure 19 show the real-time measured values of the ultra-wideband MIMO antenna offered in an indoor setting. In addition, the received power was evaluated at different distances from USRP 1 together with the MIMO 1 antenna to USRP 2 together with the MIMO 2 antenna. The distances between each of the antennas were maintained at 0.5, 0.7 and 1.0 m, and the same were chosen to avoid coupling and interference. USRPs had varying separations (0.5 mm, 0.7 mm, and 1 mm) for all 4 GHz states. Table 3 shows that all states for these distances have an RSRP of between −30 dBm and −40 dBm. The proposed MIMO antenna can behave as a transmitter as well as a receiver because of the eight radiators’ similar behavior. This makes the designed UWB MIMO antenna suitable for indoor MIMO applications.

## 5. Conclusions

The orthogonal array of the antenna elements seems to be the most practical way to reduce mutual interaction between ultra-wideband MIMO antennas, thus achieving compact dimensions. Successfully achieved mutual coupling of the suggested antenna has been below −35 dB. The 1.6 mm thick substrate was utilized to create a radiating antenna with a small footprint that is suited for diversity applications. The antenna’s dimensions are 35 × 35 mm^2^. The proposed MIMO antenna’s real-time short-range transmission and reception were tested in an indoor setting utilizing the NI 802.11 framework. With NI USRP, the proposed compact UWB MIMO antenna’s real-time performance was experimentally tested in a closed environment.

## Figures and Tables

**Table 1:** Geometrical Value of Proposed Monopole Ultra-Wideband Unit-Cell Antenna.

**Parameters**	$x_{1}$	$y_{1}$	$x_{2}$	$y_{2}$	$r_{1}$	$r_{2}$	L	$l_{1}$	$l_{2}$	$l_{3}$	$l_{g}$	$l_{s}$	W	$w_{1}$	$w_{2}$	$w_{3}$	$w_{g}$	$w_{s}$
Units (mm)	7.5	1.4	7	1	2	1.5	18	9	2	15	7	1	16	3	1	2	16	7

**Table 2:** Comparison with designs in existing literature.

Ref	Ports	Size (mm^2^)	Isolation (dB)	TARC (dB)	ECC	CCL (Bits/Sec/Hz)	GHz
[12]	4	45 × 45	>17	-	<0.02	-	3.1–13.1
[27]	4	40 × 40	>17	-	<0.03	-	2.94–14
[28]	4	39 × 39	>22	<−20	<0.02	<0.2	2.3–13.7
[29]	4	70 × 41	17	<−9	<0.012	<0.4	3.1–12
[30]	4	45 × 45	>16	-	<0.02	-	3.1–11
Prop	4	35 × 35	>35	<−10	<0.001	<0.4	3.1–11

**Table 3:** RSRP in 802.11 Framework Setting.

Mode	TxR	RxR	Distance (m)	Received Power (dBm)
State 1	P, Q, R, S	W, X, Y, Z	0.5	−30
	P, Q, R, S	W, X, Y, Z	0.7	−35
	P, Q, R, S	W, X, Y, Z	1	−40
State 2	P, Q, W, X	R, S, Y, Z	1	−40
State 3	W, X, Y, Z	P, Q, R, S	1	−40

## Data Availability

The data presented in this study are available through email upon request to the corresponding author.
